# Mitochondrial dynamics in Parkinson's disease: a role for α-synuclein?

**DOI:** 10.1242/dmm.026294

**Published:** 2017-09-01

**Authors:** Victorio M. Pozo Devoto, Tomas L. Falzone

**Affiliations:** 1 Instituto de Biología Celular y Neurociencias, IBCN (UBA-CONICET), Facultad de Medicina, Universidad de Buenos Aires, Paraguay 2155, Buenos Aires, CP1121, Argentina; 2 International Clinical Research Center (ICRC), St. Anne's University Hospital, CZ-65691, Brno, Czech Republic; 3 Instituto de Biología y Medicina Experimental, IBYME-CONICET, Vuelta de Obligado 2490, Buenos Aires, CP1428, Argentina

**Keywords:** Parkinson's disease, Synuclein, Mitochondria, Fusion-fission, Transport, Mitophagy

## Abstract

The distinctive pathological hallmarks of Parkinson's disease are the progressive death of dopaminergic neurons and the intracellular accumulation of Lewy bodies enriched in α-synuclein protein. Several lines of evidence from the study of sporadic, familial and pharmacologically induced forms of human Parkinson's disease also suggest that mitochondrial dysfunction plays an important role in disease progression. Although many functions have been proposed for α-synuclein, emerging data from human and animal models of Parkinson's disease highlight a role for α-synuclein in the control of neuronal mitochondrial dynamics. Here, we review the α-synuclein structural, biophysical and biochemical properties that influence relevant mitochondrial dynamic processes such as fusion-fission, transport and clearance. Drawing on current evidence, we propose that α-synuclein contributes to the mitochondrial defects that are associated with the pathology of this common and progressive neurodegenerative disease.

## Abstract (precis)

**Summary:** The authors review the α-synuclein structural, biophysical and biochemical properties that influence relevant mitochondrial physiological processes such as fusion-fission, transport and clearance, and propose that α-synuclein contributes to the mitochondrial defects that are associated with Parkinson's disease.

## Introduction

Parkinson's disease (PD) is the second most common neurodegenerative disorder affecting humans, with a prevalence of around 0.3% among the worldwide population (Pringsheim et al., 2014). Bradykinesia, rigidity, resting tremor and postural instability are the four cardinal motor symptoms that are induced by the slow and progressive death of dopaminergic (DA) neurons from the substantia nigra, a mesencephalic structure of the basal ganglia motor circuit (Wood-Kaczmar et al., 2006; Shulman et al., 2011). The histopathology of PD is also characterized by the presence in neurons of Lewy bodies, which are composed mainly of aggregates of the α-synuclein (α-Syn) protein (see Box 1 for a glossary of terms) (Lewy, 1912; Spillantini et al., 1998; Shults, 2006). (For an overview of the clinical features and current therapeutic strategies employed for PD, see Box 2.)

**Box 1. Glossary**

**Alpha-synuclein (α-Syn):** a small, soluble protein expressed primarily in brain tissue. Copy number variations or mutations with single amino acid exchange are associated with dominant forms of Parkinson's disease.

**Lewy bodies:** a histopathological hallmark of Parkinson's disease in the form of intracellular, insoluble inclusions, composed mainly of α-Syn among a large group of proteins.

**Mitochondrial dynamics:** group of dynamic processes that regulate mitochondrial homeostasis. These include fusion, fission, transport and mitophagy.

**Mitochondrial fusion:** the dynamic process in which two mitochondria fuse to form one elongated mitochondrion. As a result, mitochondria exchange proteins and mtDNA, leading to a renewal of molecular components. Mitofusin (Mfn) in the outer mitochondrial membrane and optic dominant atrophy (OPA1) in the inner mitochondrial membrane are the chief proteins involved in fusion.

**Mitochondrial fission:** the dynamic process in which one mitochondrion divides into two. Dynamin-related GTPase protein (Drp1) and mitochondrial fission (Fis1) are the main proteins responsible for fission. Mitochondrial fission enhances the clearance and transport of this organelle.

**Anterograde transport:** in neurons, the transport of molecular-motor-driven cargo (proteins and organelles) from the cell body (soma) to the synapse. The main proteins involved in mitochondria anterograde transport are the heavy-chain subunit of the kinesin-1 molecular motor (KIF5) with TRAK and MIRO as adaptors.

**Retrograde transport:** in neurons, the transport of molecular-motor-driven cargo (proteins and organelles) from the synapse to the soma. The main driving force for mitochondrial retrograde transport is provided by the dynein complex.

**Mitophagy:** specific process of autophagic degradation of mitochondria in lysosomes. It is mediated by the microtubule-associated proteins 1A/1B light chain 3A (LC3) in the autophagosome, which interact with p62 in the mitochondria.

**Autophagy:** intracellular degradation system in which cytoplasmic constituents are delivered to the lysosome for hydrolysis. Macroautophagy relies on engulfing large cytoplasmic components in a membrane-bound vesicle (autophagosome) for degradation. On the other hand, chaperone-mediated autophagy (CMA) involves the degradation of cytosolic proteins by their translocation into lysosomes through transporters such as the lysosome-associated membrane protein type 2A (LAMP2A).

**Box 2. Parkinson's disease: clinical features and treatment**

Parkinson's disease (PD) is a neurodegenerative disease characterized by motor impairments and the progressive death of dopaminergic (DA) neurons in the substantia nigra pars compacta. The clinical features can be divided into motor and non-motor symptoms. The motor defects include rigidity, bradykinesia, resting tremor and postural instability; non-motor symptoms include depression, dementia, hallucinations, REM sleep disorders, autonomic dysfunction and olfactory impairments. These non-motor symptoms become increasingly prevalent during the course of the disease and can be an important determinant of overall disability (Poewe, 2006). Most PD therapeutic treatments aim to ameliorate the motor symptoms, but they do not slow disease progression or tackle the cause of disease. The most widely used treatment involves the chronic administration of L-DOPA, precursor of the neurotransmitter dopamine, together with a peripheral decarboxylase inhibitor (Cotzias et al., 1969). Deep brain stimulation (DBS) has been brought into use recently for the treatment of pharmacotherapy-resistant PD (Kringelbach et al., 2007). This involves the implantation of electrical stimulators in the subthalamic nucleus to reduce motor fluctuations and tremor. Several other new therapies are in clinical trials, including caffeine, nicotine, isradipine and active or passive immunotherapy (Schneeberger et al., 2016; Oertel and Schulz, 2016). Cell therapy via the grafting of fetal mesencephalic neurons into the striatum of PD patients has been tried, but with inconclusive results so far (Petit et al., 2014). Nowadays, neurons derived from iPSCs of PD patients (familial or sporadic) are being used to analyze phenotypic abnormalities, intracellular pathways relevant for disease, cell response under stress challenges and as platforms for drug screening. Furthermore, autologous replacement therapies using neurons derived from iPSCs of patients could provide the possibility to restore neuron loss in the absence of immunocompatibility issues.

The gene coding for α-Syn, *SNCA*, was the first locus identified that linked genetics with PD (Polymeropoulos et al., 1997). Mutations in *SNCA* give rise to dominant early-onset PD (Corti et al., 2011). Owing to its presence in Lewy bodies, α-Syn research has mainly focused on its aggregation properties, and oligomerization and aggregation of α-Syn have been considered the main cause of neuronal degeneration (Melki, 2015). However, the toxicity of Lewy bodies is under debate (Jellinger, 2012). The spreading of Lewy bodies from the peripheral to the central nervous system (CNS) correlates with clinical manifestations (Braak et al., 2004), but how these observations explain the pathogenic mechanism of other genes associated with PD is not known. Currently, more than 20 loci have been genetically linked to PD, with variable penetrance, through inheritance mechanisms that range from dominant to recessive mutations or modulators of risk with small odds ratios (de Lau and Breteler, 2006; Bonifati, 2014). Mutations in these genes lead to a similar pathology and to the accumulation of α-Syn, even if the clinical manifestations do not develop in a similar way (e.g. age of onset, severity of the symptoms) (Corti et al., 2011). Thus, Lewy bodies may be considered as epiphenomena, whereas other pathogenic processes are responsible for inducing neurodegeneration.

Different studies have linked the function of α-Syn to the maintenance of mitochondrial fusion-fission, transport and mitophagy (Box 1) (Choubey et al., 2011; Nakamura et al., 2011; O'Donnell et al., 2014; Zaichick et al., 2017). These processes, considered together as mitochondrial dynamics (Box 1), are extremely relevant in neurons owing to their high morphological polarization (reviewed extensively in Itoh et al., 2013; Mishra and Chan, 2016). Interestingly, other PD-linked mutations affect genes that encode proteins with specific functions in mitochondrial dynamics (Box 3). Mutations in PTEN-induced putative kinase 1 (PINK1), Parkin, DJ-1, leucine-rich repeat kinase 2 (LRRK2) and vacuolar protein sorting-associated protein 35 (VPS35) highlight the relevance of mitochondrial dysfunction as a primary cause of neuronal death in PD (Court and Coleman, 2012; Park et al., 2014; Tang et al., 2015a,b). The involvement of mitochondrial defects in PD pathology is also supported by pharmacological evidence, which links the deleterious effects of inhibitors of the mitochondrial electron transport chain (ETC), such as 1-methyl-4-phenyl-1,2,3,6-tetrahydropyridine (MPTP), rotenone or paraquat, with DA neuronal dysfunction (Nicklas et al., 1985; Franco et al., 2010). In light of this, it is tempting to speculate that a function of α-Syn may converge with other proteins in a pathway that includes the control of mitochondrial dynamics.

**Box 3. Parkinson's disease-associated proteins in mitochondrial homeostasis**

**DJ-1:** a short ubiquitous protein with chaperone activity. Protects cells from oxidative stress and mitochondrial injury (Blackinton et al., 2009; Pantcheva et al., 2014). DJ-1 partners with PINK1 and Parkin to regulate mitochondrial homeostasis (Xiong et al., 2009). Under increased oxidative stress, DJ-1 translocates to mitochondria and regulates mitophagy (Krebiehl et al., 2010; Gao et al., 2012).

**LRRK2:** a cytosolic protein with GTPase and kinase domains (Biskup et al., 2006). Regulates mitochondrial fission by the phosphorylation of Drp1 (Wang et al., 2012; Su and Qi, 2013). LRRK2 has been found associated to the OMM (Biskup et al., 2006) and this association correlates with an increase in mitophagy (Plowey et al., 2008; Tong et al., 2010). α-Syn accumulation occurs in the presence of pathogenic mutations of LRRK2 because of impairment of CMA, a chief pathway for α-Syn degradation (Orenstein et al., 2013).

**Parkin:** an E3 ubiquitin ligase, mediates ubiquitination of several proteins with mitochondrial localization signals (Shimura et al., 2000). This process is driven by PINK1 activation, which promotes Parkin translocation to mitochondria for the control of: (i) mitochondrial fission by ubiquitination of Mfn proteins (Ziviani et al., 2010); (ii) mitochondrial anterograde transport by ubiquitination of MIRO1 (Wang et al., 2011); and (iii) mitophagy via the ubiquitination of mitochondrial proteins (Narendra et al., 2008). Mitochondrial size defects can be rescued by Parkin overexpression in α-Syn-overexpression models of PD in *Drosophila* (Haywood and Staveley, 2004). Moreover, PINK1 and Parkin mutations lead to the accumulation and aggregation of α-Syn, suggesting that these proteins have a role in α-Syn degradation (Shaltouki et al., 2015).

**PINK1:** a kinase with a mitochondrial targeting signal, stabilized in the OMM under mitochondrial depolarization conditions to control fusion-fission, transport and mitophagy (Ziviani et al., 2010; Seibler et al., 2011). PINK1 promotes mitochondrial fission through the indirect activation of Drp1 (Pryde et al., 2016). Phosphorylation of the Rho GTPase MIRO1 by PINK1 reduces the anterograde transport of mitochondria (Wang et al., 2011) and favours mitophagy (Wood-Kaczmar et al., 2008; Seibler et al., 2011). Mitochondrial fragmentation driven by α-Syn can be reduced by PINK1 overexpression (Kamp et al., 2010), whereas PINK1 mutations lead to mitochondrial dysfunction and α-Syn aggregation (Sánchez-Danés et al., 2012; Reinhardt et al., 2013; Shaltouki et al., 2015).

**VPS35:** a component of the retromer cargo-recognition complex. Regulates mitochondrial fusion by decreasing the levels of mitochondrial ubiquitin ligase 1, which promotes Mfn2 degradation ([Bibr DMM026294C162][Bibr DMM026294C163]). It also interacts and controls Drp1 turnover (Wang et al., 2016). VPS35 mutations impair the lysosomal/autophagy pathway, which enhances the α-Syn protein load, leading to increased aggregation (Tang et al., 2015a; Follett et al., 2016).

Here, we review the current knowledge in support of α-Syn localization to mitochondria, and the effects of this on mitochondrial size, distribution and clearance. Based on this evidence, we propose that α-Syn mutations or changes in its abundance contribute to mitochondrial dysfunction in PD and thus to a key pathogenic mechanism in this common neurodegenerative disease.

## α-Syn structure and function

**Fig. 1.**
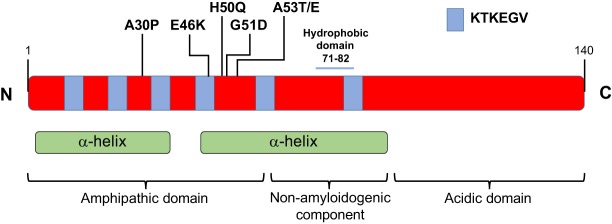
**α-Synuclein protein structure.** α-Synuclein (α-Syn) is a 140-amino-acid protein composed of three domains: the N-terminal amphipathic domain, the non-amyloidogenic component (which includes a hydrophobic core) and the C-terminal acidic domain. KTKEGV imperfect repeats span through the amphipathic domain and the non-amyloidogenic component of human α-Syn. These repeats together with the non-amyloidogenic component are responsible for the formation of two α-helices that interact with lipids. PD-related mutations (A30P, E46K, H50Q, G51D, A53T and A53E) are all located in the amphipathic domain.

*SNCA* codes for α-Syn, a small (140 amino acid) cytosolic protein that has three different domains: the N-terminal amphipathic domain; the non-amyloidogenic component (NAC) hydrophobic mid-region; and the acidic domain in the carboxyl tail (Fig. 1). The N-terminal region consists of six imperfect repeats (KTKEGV) that form two α-helix structures when the protein interacts with lipids (Ulmer et al., 2005). The middle hydrophobic region of α-Syn provides the oligomerization properties that drive the formation of the pathological Lewy body inclusions found in PD (Giasson et al., 2001). Negative charges in the C-terminal acidic domain contain chaperone-like properties that support the regulation of the oligomerization process (Li et al., 2005).

PD-associated mutations in *SNCA* occur within the N-terminal region: A30P, A53T, A53E, E46K, H50Q and G51D (Polymeropoulos et al., 1997; Kruger et al., 1998; Zarranz et al., 2004; Kiely et al., 2013; Appel-Cresswell et al., 2013; Pasanen et al., 2014) (Fig. 1). Of these, A53T and E46K cause earlier onset and more severe manifestations, whereas A30P induces late-age onset and milder symptoms (Krüger et al., 2001; Zarranz et al., 2004; Puschmann et al., 2009). Interestingly, the genomic duplication or triplication of *SNCA* provides a correlation between α-Syn expression levels and the severity of PD symptoms. Patients with an *SNCA* triplication have increased pathology, earlier onset and acute progression compared to patients with an *SNCA* duplication (Fuchs et al., 2007). In addition, polymorphisms in the promoter region of *SNCA* that increase α-Syn expression levels have been associated with a higher risk of developing PD (Hadjigeorgiou et al., 2006).

α-Syn accounts for 1% of the total protein content in neurons, with an enriched presynaptic localization (Iwai et al., 1995). It is also reported to be widely distributed within neurons, being present in the cytosol, nucleus, mitochondria and the mitochondria-associated membranes (MAMs) (Maroteaux et al., 1988; Li et al., 2007; Guardia-Laguarta et al., 2014). Many physiological and pathological functions have been proposed for α-Syn. These include: (i) a role in regulating vesicle fusion and neurotransmitter release (Abeliovich et al., 2000; Liu et al., 2004), which is supported by the interaction of α-Syn with the presynaptic soluble NSF attachment protein receptor (SNARE) complex (Burré et al., 2010); (ii) a role in intracellular trafficking, which is supported by impaired vesicular transport between the endoplasmic reticulum (ER) and Golgi when α-Syn is overexpressed in neurons (Cooper et al., 2006); (iii) a role in the regulation of cell death, due to the protective and antiapoptotic effects of α-Syn in the presence of caspase activation (Alves Da Costa et al., 2002); and (iv) a role in protein clearance, since mutant α-Syn inhibits lysosomal degradation by binding to lysosome-associated membrane glycoprotein 2A (LAMP2A) and blocking protein uptake (Cuervo et al., 2004). In addition, α-Syn can oligomerize to form fibrils, which have been shown to spread from cell to cell; this is the most extensively studied characteristic of α-Syn and is thought to contribute to its pathogenicity (Desplats et al., 2009; Frost and Diamond, 2010; Lee et al., 2010b; Winner et al., 2011; Lashuel et al., 2013; Prusiner et al., 2015). α-Syn has also been proposed to regulate different processes associated with the maintenance of neuronal mitochondrial homeostasis (Kamp et al., 2010; Nakamura et al., 2011; Pozo Devoto et al., 2017). In the next section we introduce the processes involved in mitochondrial dynamics and address the current evidence that supports a role for α-Syn as a modulator of these processes.

## A role for α-Syn in mitochondrial dynamics

**Fig. 2.**
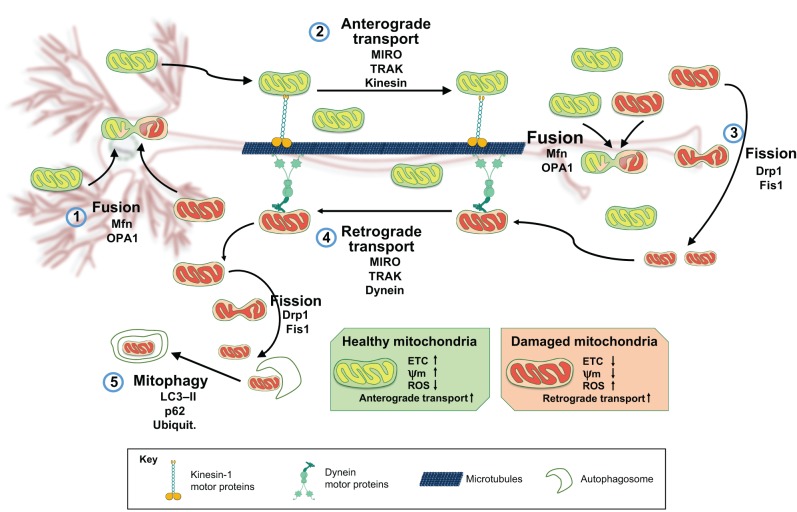
**Neuronal mitochondrial dynamics.** A schematic of a neuron is shown in the background, with the cell body (soma) and dendrites (left), and axon (centre). Mitochondrial dynamics in neurons is orchestrated by regulated rates of mitochondrial fusion and fission, transport and mitophagy. Damaged mitochondria (red) can be restored by fusion (1) with healthy mitochondria (green), a process that mainly occurs in the neuronal soma and is driven by Mfn and OPA1. Healthy mitochondria are transported along the axon from soma to synapses by the anterograde axonal transport system (2), which delivers mitochondria to distant locations (proteins involved in this system are MIRO, TRAK and the kinesin-1 family of molecular motors). In neurons, mitochondria respond to internal and external needs by modulating the rates of fusion and fission. Aged or damaged mitochondria (red) can undergo fission (3) driven by Drp1 and Fis1, and are taken back to the soma by the retrograde axonal transport machinery (4) (proteins involved in this system are MIRO, TRAK and the molecular motor dynein). Once these mitochondria reach the cell body, they may be cleared by mitophagy (5) (p62 and LC3-II are involved in regulating this process). ETC, electron transport chain; Mfn, mitofusin; OPA1, optic dominant atrophy 1; MIRO, mitochondrial Rho GTPase; ROS, reactive oxygen species; Ψ_m_, mitochondrial membrane potential; TRAK, trafficking kinesin protein; Kinesin, kinesin heavy chain 5A-C; Drp1, dynamin-related protein 1; Fis1, mitochondrial fission 1; LC3-II, microtubule-associated proteins 1A/1B light chain 3A.

Within a neuron, the soma, synapse and nodes of Ranvier have high energy requirements (Sheng and Cai, 2012). Several processes are involved in the maintenance of mitochondrial dynamics to ensure that these high energy requirements can be met, namely: fusion-fission, transport and mitophagy (Fig. 2). Each of these processes is interconnected via a complex relationship that maintains a functional mitochondrial network throughout the neuron and during its lifetime. Many mitochondrial proteins interact closely and modulate the number and distribution of neuronal mitochondria (Frederick and Shaw, 2007; Kageyama et al., 2011). Mitochondrial functions in different neuronal regions include the buffering of presynaptic Ca^2+^, the maintenance of membrane potential, and the provision of energy for axonal transport and for neurotransmitter uptake and recycling (Schon and Przedborski, 2011). These functions are challenging for DA neurons owing to their polarization and extensive arborization of axonal projections (Hunn et al., 2015). DA neurons are also subjected to higher loads of reactive oxygen and nitrogen species (ROS and RNS, respectively) as a result of DA biosynthesis (Rodriguez et al., 2015). Mitochondria are the main source (90%) of ROS, and increased ROS levels as a consequence of mitochondrial dysfunction might compromise DA neuron survival (Guzman et al., 2010; Perfeito et al., 2012). Thus, subtle defects in mitochondrial dynamics could be a slow but steady factor that affects mitochondrial homeostasis in a sensitive cell population (Rubinsztein et al., 2005; Amiri and Hollenbeck, 2008; Becker et al., 2009; Zhang et al., 2014).

A recent characterization of mitochondria in neurons from *Caenorhabditis elegans* revealed a mitochondria size increase during development, followed by a steady maintenance and a progressive decline of size and density during the organism's lifespan (Morsci et al., 2016). These stages depend on fusion-fission proteins and on changes in mitochondrial transport and clearance, highlighting the influence of these processes in neuronal ageing, which is also the main risk factor for PD (Collier et al., 2011; Niccoli and Partridge, 2012). Whether mitochondrial defects cause PD or occur as a consequence of this disease remains to be elucidated (Wang and Hekimi, 2015).

### Does α-Syn localize to mitochondria?

In addition to its presence in the cytosol and nucleus of neurons, α-Syn has been shown to localize to mitochondria in a wide range of experimental models (Martin et al., 2006; Li et al., 2007; Devi et al., 2008; Cole et al., 2008; Shavali et al., 2008; Chinta et al., 2010; Kamp et al., 2010). The presence of endogenous α-Syn in mitochondria has been reported in neurons in mice (Li et al., 2007; Zhang et al., 2008). Moreover, a study showed that α-Syn levels in mitochondrial fractions are similar to its levels in other synaptic-derived membranes or vesicle fractions obtained from mouse brains (Nakamura et al., 2008). Interestingly, mitochondria in the substantia nigra of post-mortem brains from PD patients were found to be enriched for α-Syn (Devi et al., 2008; Devi and Anandatheerthavarada, 2010). However, the exact localization of α-Syn within mitochondria remains unclear. Different immunogold electron microscopy studies have reported that α-Syn could be present in the outer mitochondrial membrane (OMM) (Cole et al., 2008; Kamp et al., 2010), in both the OMM and inner mitochondrial membrane (IMM) (Li et al., 2007; Devi et al., 2008), and in the mitochondrial matrix (Zhang et al., 2008). In a study of PD post-mortem human brains in which the OMM was biochemically stripped, the authors reported that α-Syn localizes to the IMM (Devi et al., 2008). Given that α-Syn lacks a true mitochondrial localization signal, the mechanism underlying its internalization remains unclear. It has also been shown that most of the membrane-bound α-Syn does not localize to mitochondria but to MAMs (Guardia-Laguarta et al., 2014), hinting at a possible, as yet unexplored, MAM compartment that might be relevant for understanding the role of α-Syn in PD pathogenesis.

The mitochondrial localization of α-Syn can be further enhanced by its overexpression and is also influenced by its pathogenic mutations (Shavali et al., 2008; Kamp et al., 2010). Distinct pathogenic variants that contain a single amino-acid substitution in the N-terminal domain display different affinities for membranes and for mitochondrial localization. For example, the A53T α-Syn variant is highly enriched in isolated mitochondria when compared to the wild-type (WT) protein or A30P variant (Devi et al., 2008). Moreover, the translocation of A30P to mitochondria was shown to be reduced in a cellular model of elevated oxidative stress (Cole et al., 2008), and we recently confirmed the enrichment of A53T α-Syn and relatively low levels of A30P α-Syn in mitochondrial fractions when overexpressed in the human neuroblastoma cell line SHSY5Y (Pozo Devoto et al., 2017). However, another recent study reported reduced levels of A30P and A53T variants in crude mitochondrial fractions from the M17 neuroblastoma line when compared to WT α-Syn levels (Guardia-Laguarta et al., 2014). Altogether, there is solid evidence supporting α-Syn localization to mitochondria; however, it remains to be elucidated how different α-Syn mutations can modulate its localization. In the next section we present the biophysical properties of α-Syn that mediate its association with membranes and may support the interaction with mitochondria.

### The molecular basis for an α-Syn–mitochondria interaction

In contrast to its intrinsically disordered state in solution, α-Syn adopts a highly helical conformation when associated with lipids (Fig. 1). When any of the three exons encoding its N-terminal domain are deleted, the interaction between α-Syn and vesicles is disrupted (Perrin et al., 2000). These deletions also reduce the presynaptic localization of α-Syn, suggesting that its reduced association with vesicles also impairs its transport along axons (Yang et al., 2010). Further proof that the N-terminus of α-Syn associates with membranes comes from the protection of its residues 1 to 103 from proteolytic cleavage when α-Syn is incubated with sodium dodecyl sulfate (SDS) micelles or with lipid vesicles (Bussell and Eliezer, 2004).

Pathogenic mutations in α-Syn, which all occur in the first or second helical regions, induce changes in its lipid-binding properties. The A30P mutation disrupts the first α-helical region, reducing its affinity for lipids in different experimental models (Jensen et al., 1998; Jo et al., 2002; Bussell and Eliezer, 2004; Perlmutter et al., 2009; Bodner et al., 2010). Conversely, the A53T mutation induces no change or even increases the membrane affinity of α-Syn, generating new intra-protein hydrogen bonds that stabilize this configuration (Jensen et al., 1998; Perrin et al., 2000; Jo et al., 2002; Bussell and Eliezer, 2004; Perlmutter et al., 2009; Bodner et al., 2010). The E46K mutation eliminates a negative charge present in WT α-Syn, thereby removing a repulsive interaction that might explain the higher affinity of E46K mutant protein for vesicles that contain negatively charged lipids (Perlmutter et al., 2009; Bodner et al., 2010). The interaction of α-Syn with membranes supports its association with a membrane-bound organelle such as mitochondria, and this association could be modulated by changes to its N-terminal sequence.

In *in vitro* studies, the binding of α-Syn to large unilamellar vesicles correlates with the concentration of the non-bilayer-forming lipid cardiolipin (CL), a type of lipid enriched in mitochondrial membranes (Zigoneanu et al., 2012; Horvath and Daum, 2013; Robotta et al., 2014). Another study has shown that the binding of α-Syn to intact mitochondria can be reduced using nonyl acridine orange (a CL-binding competitor), and that stripping external proteins from mitochondria has no effect on the association between α-Syn and mitochondria (Cole et al., 2008). This suggests that this association is not protein dependent (Cole et al., 2008). The protonation of α-Syn under acidic intracellular pH also increases its binding to mitochondrial membranes, and this effect can be explained by the high content of acidic phospholipids in mitochondria (Cole et al., 2008; Nakamura et al., 2008).

Protein-protein interactions have also been proposed to mediate the association of α-Syn with mitochondria. Distinct mitochondrial protein complexes, such as the transmembrane voltage-dependent anion channel (VDAC) (Martin et al., 2014), translocase of the outer membrane 40 (TOM40) (Bender et al., 2013) or the protein transporter TOM20 (Di Maio et al., 2016), are candidates for interacting with α-Syn and have been proposed to mediate its internalization. α-Syn has differential affinity for VDAC, which is located in the OMM (Martin et al., 2014). This affinity is based on the anionic C-terminal region of α-Syn, and depends on the electrical potential displayed by the mitochondrial membrane (Rostovtseva et al., 2015). Another study supports the α-Syn translocation in mitochondria through a TOM40-dependent mechanism, based on the observation of reduced α-Syn in mitochondria after the specific blockage of TOM40 with antibodies (Devi et al., 2008). Transgenic mice overexpressing WT or A53T α-Syn show a marked decrease of TOM40 protein expression with age, a phenotype that is not present in A30P-overexpressing mice; however, the mechanism for this change of expression is still unknown (Bender et al., 2013). Recently, a high-affinity interaction between α-Syn and TOM20 was reported and proposed to lead to impaired mitochondrial respiration when α-Syn is overexpressed (Di Maio et al., 2016). In addition, a reduction in mitochondrial protein import through TOM20 has been correlated with an increased α-Syn–TOM20 interaction observed in PD human brains (Di Maio et al., 2016). The active translocation of α-Syn into mitochondria appears to depend on mitochondrial membrane potential and on oxidative phosphorylation activity. This is supported by the finding that carbonyl cyanide *m*-chlorophenyl hydrazone (CCCP) or oligomycin, acting as ionophore or oxidative phosphorylation inhibitors, respectively, reduced the incorporation of α-Syn into isolated rat liver mitochondria (Devi et al., 2008). How different mutations affect the ability of α-Syn to bind to these proteins remains to be addressed.

These two alternative models of α-Syn–mitochondria interaction via lipids or proteins may also act cooperatively to increase the localization of α-Syn in the OMM, the IMM and the matrix. A role for α-Syn in the modulation of mitochondrial dynamics is supported by its structural properties that enable the interaction with intracellular membranes, the finding that almost all α-Syn mutations lie within its membrane-interaction domain and the identification of mitochondrial proteins that associate with α-Syn.

### α-Syn role in mitochondrial fusion and fission

It remains to be shown that α-Syn directly controls mitochondrial morphology; however, its overexpression leads to mitochondrial fragmentation, as has been reported in *C. elegans* (Kamp et al., 2010; Butler et al., 2012), dorsal root ganglia neurons of zebrafish (O'Donnell et al., 2014), HeLa immortalized cells (Nakamura et al., 2011), human embryonic kidney (HEK) immortalized cells (Butler et al., 2012), the SH-SY5Y neuroblastoma cell line and transgenic mice expressing the human α-Syn A53T mutation (Xie and Chung, 2012). Furthermore, it has recently been shown that the forced delivery of α-Syn to the mitochondria through a protein dimerization assay can increase the mitochondrial fragmentation phenotype in human neurons (Pozo Devoto et al., 2017).

**Fig. 3.**
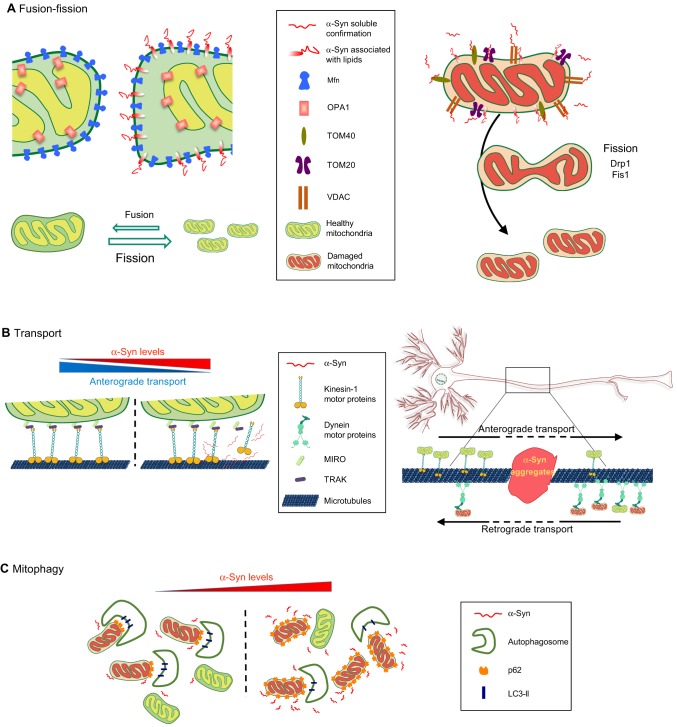
**α-Syn in mitochondrial dynamics.** A schematic highlighting the putative pathogenic roles of α-Syn in key processes involved in mitochondrial homeostasis: fusion, fission, transport and mitophagy. (A) Fusion-fission: overexpression of wild-type (WT) or mutated α-Syn increases its localization to the outer mitochondrial membrane (OMM). This interaction of α-Syn with lipids reduces membrane curvature, thus reducing fusion and pushing the balance towards fission (left panel). α-Syn is thought to interact with VDAC, TOM20 and TOM40, disrupting their function and leading to induction of mitochondrial fission (right panel) (see main text for details). (B) Transport: mitochondria (healthy and damaged) are transported along the axon between the soma and synapses of neurons via anterograde and retrograde transport (see Box 1 and Fig. 2). Overexpressed α-Syn may sequester or reduce the function of the molecular motors involved, decreasing the anterograde transport of mitochondria (left panel). α-Syn oligomerization or aggregation in axons may physically impair the transport of mitochondria (right panel). (C) Mitophagy: damaged mitochondria are cleared by mitophagy (see Box 1 and Fig. 2). α-Syn overexpression is thought to reduce levels of LC3-II and decrease autophagosome formation, resulting in the accumulation of mitochondria tagged by p62 for mitophagy (see main text for details).

Mitochondrial morphology is highly regulated, and this regulation is mediated by interactions between the proteins that control mitochondrial fusion and fission (Burté et al., 2015). Under physiological conditions, mitochondrial fusion is considered a process that favours biogenesis by the exchange of new protein and mitochondrial DNA between the merging organelles. Fusion restores functional proteins and non-damaged mitochondrial DNA to dysfunctional mitochondria, and this process decreases the occurrence of mitophagy (Itoh et al., 2013) (Fig. 2). The molecular process of fusion is driven by mitofusins 1 and 2 (Mfn1 and Mfn2), proteins from the dynamin-related GTPase family (Meeusen et al., 2004). Mitofusins in the OMM and optic dominant atrophy 1 (OPA1) in the IMM work in collaboration to sequentially fuse the mitochondrial membranes (Song et al., 2009). Counterbalancing fusion is the fission process, which reduces mitochondrial size, leading to enhanced mitochondrial axonal transport and mitophagy (Itoh et al., 2013). Fission is led by the activity of the dynamin-related GTPase protein (Drp1), which is recruited to mitochondria by receptor-like proteins: mitochondrial fission factor (Mff) and mitochondrial fission 1 (Fis1). Once attached to the OMM, Drp1 forms a ring-shaped structure that acts as a constricting diaphragm in the mitochondrial membrane (Burté et al., 2015) (Fig. 3). In addition to these essential regulatory proteins, the lipid composition in the mitochondrial membrane can also modulate the rates of fusion and fission of this organelle (Joshi et al., 2012; Chan and McQuibban, 2012; Zhang et al., 2014).

The relevance of mitochondrial fusion and fission to PD pathology is highlighted by the drastic deleterious effect on nigrostriatal connections that is induced by the conditional deletion of mitochondrial membrane proteins in DA neurons of mice. The deletion of *Mfn2* induces aberrant mitochondrial morphologies and impairs ETC activity, with a severe loss of nerve terminals in the striatum (Lee et al., 2012; Pham et al., 2012). *Drp1* deletion in mice causes the depletion of mitochondria in the axons and terminals of the striatum, followed by axonal dieback and DA neuronal death (Berthet et al., 2014). Although there is no direct association known between the loss of core fusion-fission proteins and PD, other neurodegenerative diseases such as Charcot-Marie-Tooth 2A, dominant optic atrophy 1 and postneonatal death with neurodevelopmental disorders are induced by *Mfn2*, *Opa1* or *Drp1* mutations, in respective order (Burté et al., 2015).

The mechanism that underlies the α-Syn-mediated changes in mitochondrial morphology is not clear, but recent findings shed some light on this question. Mitochondrial size varies in different α-Syn models: A53T mutants shows the most notable fragmentation effect, followed by mild fragmentation induced by WT α-Syn overexpression, whereas almost no fragmentation is observed in the presence of the A30P variant (Nakamura et al., 2011; Butler et al., 2012; Xie and Chung, 2012; Gui et al., 2012; Pozo Devoto et al., 2017). However, these phenotypes are not always consistent, since other studies have reported that A30P α-Syn induces similar or even greater fragmentation defects to A53T (Kamp et al., 2010; Guardia-Laguarta et al., 2014). The interaction of α-Syn with lipids creates changes in the membrane structure. When α-Syn is bound to SDS micelles, a reduction in the membrane curvature is induced (Perlmutter et al., 2009), and the same occurs when it is in association with lipid bilayers (Braun et al., 2012). Furthermore, the fusion of small, unilamellar vesicles *in vitro* is negatively correlated with increasing concentrations of α-Syn, which suggests that the α-Syn effect on membrane curvature could be responsible for reducing vesicle fusion dynamics (Chernomordik et al., 1995; Kamp et al., 2010). The N-terminal fragment and the NAC region of α-Syn are necessary and sufficient to modify membrane curvature, showing that this property arises from its lipid-interacting domain (Kamp et al., 2010). If this is the case, the binding of α-Syn to the OMM could affect membrane curvature and decrease the rate of mitochondrial fusion (Fig. 3), thus explaining the reduced mitochondrial size in α-Syn mutants. This is further supported by the increase in mitochondrial fragmentation when WT or A53T α-Syn are targeted to the OMM, whereas A30P α-Syn does not induce mitochondrial size changes under similar conditions (Pozo Devoto et al., 2017).

Some studies support a link between α-Syn and the proteins responsible for mitochondrial fusion and fission (Xie and Chung, 2012; Gui et al., 2012; Menges et al., 2017). Reductions in *Mfn1*, *Mfn2* and *Drp1* expression correlate with a decrease in mitochondrial size in the spinal cord of transgenic mice overexpressing A53T (Xie and Chung, 2012). Similarly, A53T overexpression in SH-SY5Y cells induced expression of *DLP* (a *Drp1* orthologue) and its translocation to mitochondria to modify mitochondrial fission (Gui et al., 2012). By contrast, α-Syn effects on mitochondrial size that are independent of fusion-fission proteins have also been described (Kamp et al., 2010; Nakamura et al., 2011; Guardia-Laguarta et al., 2014; Liu et al., 2015; Pozo Devoto et al., 2017). Mitochondrial fragmentation induced by α-Syn still occurs in fibroblasts from *Drp1* knockout mice (Nakamura et al., 2011; Guardia-Laguarta et al., 2014). α-Syn overexpression reduces the number of cells with elongated mitochondria derived from the overexpression of fusion proteins (Mfn1, Mfn2 and Opa1) (Kamp et al., 2010). Moreover, α-Syn knockdown by siRNA in *C. elegans* (Kamp et al., 2010) or its N-terminal disruption by genomic editing in human induced pluripotent stem cells (iPSCs) (Pozo Devoto et al., 2017) lead to mitochondrial elongation in neurons, suggesting that α-Syn influences mitochondrial size by acting directly on the fusion-fission process (Fig. 3).

The hypothesis that α-Syn regulates the size of neuronal mitochondria via its affinity for lipids or via protein-protein interactions is attractive and, if proven, one would expect compliance with the following statements: (i) changes in mitochondrial size should correlate with the levels of α-Syn expression; thus, the higher the concentration of α-Syn, the larger the reduction in mitochondrial size; and (ii) mutations in α-Syn that increase its membrane affinity must enhance the fragmentation phenotype, whereas mitochondrial elongation should be induced by mutations that reduce α-Syn membrane interaction. Although little is known about the progression of mitochondrial size defects in PD, the first hypothesis is supported by clinical observations: patients with *SNCA* triplications have earlier onset and more severe symptoms than those with duplications (Ibáñez et al., 2009). The A53T mutation induces earlier and more severe clinical features of PD compared to PD patients carrying the A30P mutation, who show later disease onset and usually a milder course of disease (Krüger et al., 2001; Puschmann et al., 2009). Neurons derived from reprogramming of fibroblasts of patients harbouring A53T and A30P α-Syn mutations could provide a first step to validate the relationship between endogenous levels of α-Syn variants and mitochondrial phenotypes. If the regulation of mitochondrial size somehow depends on the interaction of α-Syn with mitochondrial membranes, then impairing this size balance might have a long-term impact on neuronal homeostasis.

### α-Syn impact on mitochondrial transport processes

As discussed above, α-Syn may affect mitochondrial morphology but it remains possible that it affects morphology by impairing the mobilization of mitochondria along the axon. In neurons, the shape of mitochondria depends on their ability to move; therefore, a tight relationship exists between the morphology of mitochondria and their transport in axons (Fig. 2) (Frederick and Shaw, 2007). Impaired mitochondrial transport is induced as a consequence of protein aggregation in neurofibrillary tangles or in Lewy neurites (abnormal accumulations of α-Syn in swollen axons or dendrites) (Spillantini et al., 1998; Galvin et al., 1999). The movement of mitochondria along axons is bidirectional (Morris and Hollenbeck, 1995). The anterograde transport of mitochondria to synapses is mediated by kinesin motors and their retrograde transport to cell bodies by dynein motors (Hirokawa et al., 2010) (Box 1). The interaction of the *Drosophila* mitochondrial adaptor protein Milton (and also its mammalian orthologues, TRAK1 and TRAK2) (Macaskill et al., 2009; Koutsopoulos et al., 2010; van Spronsen et al., 2013) with mitochondrial Rho GTPase 1 (MIRO1), which is located on the OMM, mediates the anterograde transport of mitochondria by associating with the heavy-chain subunit (KIF5A, B and C) of the kinesin-1 molecular motor (Hurd and Saxton, 1996; Tanaka et al., 1998; Stowers et al., 2002; Glater et al., 2006). Mutations in KIF5 impair the localization of mitochondria and of many other cargos. By contrast, mutations in Milton induce selective mitochondrial trafficking defects (Tanaka et al., 1998; Glater et al., 2006). MIRO1, bound to the OMM through its C-terminal transmembrane domain, regulates mitochondrial transport through its Rho-GTPase activity and Ca^2+^-binding motifs that can sense intracellular Ca^2+^ concentration (Fransson et al., 2003; Frederick et al., 2004). The interaction of dynein retrograde motors with mitochondria is also mediated by MIRO1, which suggests that MIRO1 acts to regulate the transport direction of axonal mitochondria (Guo et al., 2005; Russo et al., 2009). Indeed, elevated cytosolic Ca^2+^ can arrest mitochondrial mobility through a mechanism that involves molecular motor inactivation or the disassembly of the KIF5-TRAK-MIRO1 complex (Macaskill et al., 2009). It is conceivable that proteins that transiently associate with mitochondria can recruit or impair the molecular motor machinery, thereby having a role in the regulation of mitochondrial distribution and transport.

Experimental evidence has indicated that α-Syn interferes with mitochondrial transport. α-Syn overexpression induced mitochondrial transport deficits prior to axonal degeneration in zebrafish (O'Donnell et al., 2014). Furthermore, human-derived neurons in which α-Syn is overexpressed develop early defects in anterograde-to-retrograde mitochondrial flux, suggesting a regulatory role for α-Syn on mitochondrial axonal transport (Pozo Devoto et al., 2017). To be considered to function within a pathway that regulates axonal transport, α-Syn should somehow interact with the transport machinery or, when mutated, be able to induce defects in the mitochondrial transport system. The cytosolic presence of α-Syn in neurons suggests that its distribution is not likely to be mediated via the secretory pathway (Clayton and George, 1998). However, the enrichment of α-Syn in synapses and its intrinsic membrane affinity indicate that α-Syn has a transient and regulated association with transported vesicles for its delivery to synapses (Roy et al., 2007), a mechanism that has been described in neurons for other cytosolic complexes, such as the proteasome (Otero et al., 2014). Interestingly, co-immunoprecipitation data indicate that α-Syn can interact with the molecular motor machinery, specifically with a complex containing kinesin-1 (Utton et al., 2005). This interaction may regulate the transport of other cargos, including that of mitochondria, owing to the sequestration of active motors under α-Syn overexpression (Utton et al., 2005). In addition, the presence of immobile α-Syn inclusions in axons, induced by the recruitment of α-Syn located on mobile vesicles, has been proposed to seed further α-Syn aggregation and induce selective transport defects (Volpicelli-Daley et al., 2014). Oligomeric α-Syn has also been shown to interfere directly with axonal transport by disrupting the association of kinesin-1 motors with microtubules (Prots et al., 2013). The number of active motors interacting with microtubules is proposed as a mechanism that regulates transport velocity and directionality (Leidel et al., 2012; Fu and Holzbaur, 2014; Lacovich et al., 2017). Interestingly, the velocity of microtubule gliding across kinesin-coated surfaces is significantly decreased in the presence of α-Syn oligomers (Prots et al., 2013). These data link the intrinsic aggregation properties of α-Syn to a mechanism for mitochondrial transport defects due to kinesin function impairments. Moreover, the considerable accumulation of proteins, vesicles and mitochondria within axonal swellings that are positive for α-Syn staining in sporadic PD suggest that motility defects contribute to disease pathogenesis (Spillantini et al., 1998; Galvin et al., 1999). Such mechanisms could lead to abnormal mitochondrial distribution, thereby inducing reduced mitochondrial clearance and the mitochondrial fragmentation phenotype seen in PD (Volpicelli-Daley et al., 2014). Thus, one of the ways α-Syn might regulate mitochondrial homeostasis is by modifying mitochondrial mobility. Recently, several studies have indicated that fusion-fission dynamics are linked to mitochondrial axonal transport (Fransson et al., 2003; Saotome et al., 2008; Macaskill et al., 2009; Falzone et al., 2009; Liu and Hajnóczky, 2009; Misko et al., 2010; Pham et al., 2012; Berthet et al., 2014). It is therefore possible that defects in mitochondrial function in PD occur as a consequence of their abnormal distribution and transport, which in turn could be induced by excessive levels of α-Syn or by its abnormal aggregation (Fig. 3). If future research confirms these associations, they are likely to be relevant for the pathology of PD.

### α-Syn regulation of mitophagy

Whether α-Syn impairs the clearance of mitochondria has not been proven; however, a close association between α-Syn and autophagy (Box 1) has been established by the finding that α-Syn can be degraded by chaperone-mediated autophagy (CMA) and/or macroautophagy (Vogiatzi et al., 2008). Interestingly, α-Syn aggregates compromise the autophagic mechanism by impairing the membrane-engulfing process that is needed for protein degradation in neuronal cell lines and α-Syn transgenic mice (Winslow et al., 2010). This suggests that defects in autophagy induced by α-Syn could lead to dysfunctions in mitochondrial clearance.

Mitochondrial catabolism can be divided into two main processes: the autophagic degradation of whole mitochondria in the process of mitophagy and the specific breakdown of mitochondrial contents. In the latter process, proteins and lipids bud out in mitochondrial derived vesicles (MDVs) that fuse with lysosomes, where these molecules are broken down (Ashrafi and Schwarz, 2013). During mitophagy, a whole mitochondrion is engulfed by a double-membrane body, the autophagosome. This structure then fuses with lysosomes to form the autolysosome, inside which the engulfed mitochondrion is degraded. For the engulfing process to occur, microtubule-associated proteins 1A/1B light chain 3A (LC3) in the autophagosome interact with p62 and/or CL in mitochondria (reviewed in Ashrafi and Schwarz, 2013; Lionaki et al., 2015). When mitochondrial membrane potential is lost, p62 protein is recruited to mitochondria after the ubiquitination of mitochondrial membrane proteins (Lee et al., 2010a,b). However, inhibitors of the ETC induce mitophagy through CL without the recruitment of p62. Thus, depolarizing and non-depolarizing damage to mitochondria can activate different mitophagy pathways.

α-Syn-derived impairments in autophagy have been proposed by different studies. One study reported that overexpression of α-Syn in a neuroblastoma cell line enhanced levels of the autophagic substrate p62 and led to significant decreases in levels of the autophagy regulator LC3, and in the number of LC3-II-positive vesicles (Winslow et al., 2010). A later study suggested that autophagic activity is impaired when α-Syn becomes aggregated (Tanik et al., 2013). α-Syn overexpression also has a direct inhibitory effect on Rab1 GTPase protein and causes the mislocalization of autophagy related protein 9 (ATG9) (Winslow et al., 2010). In contrast, the A53T α-Syn mutation might have a different impact on autophagy. An increase in lysosome-mediated mitophagy has been described in DA neurons of A53T transgenic mice, which could be indicative of a compensatory response to remove defective mitochondria (Chinta et al., 2010). Using a conditional A53T transgenic mouse model, impairments in the autophagosome-lysosome pathway were observed, including increased levels of lysosomal markers p62, LC3 and LAMP2A in aged DA neurons of the CNS (Lin et al., 2012). However, no abnormality of mitochondrial morphology was observed. Similar increases in mitophagy were reported after A53T overexpression in mouse cortical neurons, which also leads to neuronal death (Choubey et al., 2011). The mitochondrial phenotype can be reverted by silencing the ubiquitin ligase Parkin, and by overexpression of Mfn or a dominant-negative variant of Drp1 (Choubey et al., 2011).

α-Syn mutants, but not WT α-Syn, bind to the LAMP2 transporter in the lysosomal membrane and act as a blocker for protein uptake in the CMA pathway, thereby inhibiting their own degradation and that of other substrates (Cuervo et al., 2004). In humans, Lewy bodies are observed within intracellular regions that contain accumulations of autophagy-related proteins (Alvarez-Erviti et al., 2010). The occurrence of defects in CMA in PD is suggested by the reduced expression of LAMP2A in the substantia nigra pars compacta and the amygdala of human PD post-mortem brains, compared to age-matched controls (Alvarez-Erviti et al., 2010). Thus, α-Syn overexpression or the physiological effects of α-Syn mutations can lead via different mechanisms to impaired autophagy, which in turn could compromise the clearance of abnormal mitochondria by mitophagy (Fig. 3).

A reduction in mitochondrial size is a requirement for mitochondrial clearance. This view is supported by findings showing that elongated mitochondrial phenotypes prevent mitophagy as a result of protein kinase A (PKA)-mediated inhibition of Drp1, even during fibroblast starvation when the autophagic process should be increased (Gomes et al., 2011). Also, mitochondria arranged in large tubular masses with abnormal function and degradation have been described in mouse neurons lacking Drp1 (Kageyama et al., 2012). In line with these studies, a decrease in the mitophagy rate is observed both by FIS1 protein knock down or by the overexpression of OPA1 (Twig et al., 2008). In neurons, mitophagy is linked to the transport process owing to the need for the somatic recovery of mitochondria for clearance. Thus, even if α-Syn does not directly regulate mitochondrial clearance via autophagy, the role of α-Syn in fusion-fission and transport processes could indirectly regulate the rate of mitopaghy.

### Perspectives and future outlook

Several proteins are involved in processes that maintain mitochondrial density and distribution throughout the cell. The importance of mitochondrial dynamics to neuronal homeostasis is highlighted by the development of neuropathies in patients that harbour mutations in proteins directly involved in fusion-fission (such as Mfn, OPA1, etc.). Since the discovery that PINK1/Parkin pathway can regulate mitochondrial clearance (Yang et al., 2006; Narendra et al., 2008), more proteins associated to PD have been linked to the control of mitochondrial dynamics (Xiong et al., 2009; Ziviani et al., 2010; Wang et al., 2012, 2016; Pryde et al., 2016). Here, we have focused on α-Syn, highlighting its molecular and biophysical properties that support a physiological function in the regulation of mitochondrial dynamics. We particularly emphasized the studies that show that this function can be disrupted by changes in α-Syn dosage or structure, in *in vitro* or *in vivo* models. Moreover, the convergence of different proteins associated with PD in the regulation of mitochondrial dynamics makes this process a strong candidate for a pathogenic mechanism underlying PD.

A remaining challenge is to understand the specific mechanism of the α-Syn-mitochondria interaction, a key step that will contribute to a molecular model explaining how α-Syn mutations or dosage induce changes in the mitochondria. Interestingly, mitochondrial size correlates with the intrinsic α-Syn biophysical properties within each α-Syn variant. Upon membrane interaction, α-Syn changes mitochondrial lipid rigidity or curvature that is involved in the fusion-fission process. α-Syn also regulates the function of mitochondrial translocators, ETC proteins or fusion-fission proteins by direct protein-protein interactions. In neurons, where α-Syn expression is enriched, its physiological roles include the modulation of mitochondrial morphology, transport and clearance. Therefore, subtle changes induced by abnormal α-Syn behaviour can give rise, in the long term, to impairments in mitochondrial distribution and structure, leading to significant defects in the neuronal energy-producing organelle. Different studies in relevant disease models have centred attention on the correlation between dosage or mutations of α-Syn and changes in mitochondrial morphology or location. The long-term effect of these molecular mechanisms should be elucidated to comprehend their impact on neuronal function. Moreover, efforts should be directed to understand whether DA neurons may display differential sensitivities to α-Syn alterations compared with other neuronal types. Novel protocols to obtain highly enriched DA neurons or 3D cultures, such as organoids, are likely to become important tools with which to test DA sensitivity and glial influence on neuronal fate under PD-associated mutations. Furthermore, new technologies for cell reprogramming and neuronal differentiation in disease modelling will pave the way for understanding the role of α-Syn in mitochondrial quality control. Relevant information related to α-Syn pathogenicity is arising from cell reprogramming (Okita et al., 2011; Ran et al., 2013; Flierl et al., 2014; Pozo Devoto et al., 2017) and/or genome-editing in patient cells harbouring α-Syn, LRRK2 (Cooper et al., 2012; Su and Qi, 2013), Parkin (Jiang et al., 2012; Imaizumi et al., 2012) or PINK1 (Seibler et al., 2011; Rakovic et al., 2013) mutations.

By investigating the role of α-Syn in mitochondrial homeostasis in health and disease, we are likely to uncover new findings that shed light on the mitochondrial processes that regulate its size, distribution and clearance. Understanding better the influence of this multi-faceted PD-related protein will reveal the importance of mitochondrial dynamics for DA neuronal function and survival, and ultimately PD progression.
